# Targeting *KRAS* mutant lung cancer: light at the end of the tunnel

**DOI:** 10.1002/1878-0261.13168

**Published:** 2022-01-18

**Authors:** Matthias Drosten, Mariano Barbacid

**Affiliations:** ^1^ Molecular Oncology Program Centro Nacional de Investigaciones Oncológicas (CNIO) Madrid Spain

**Keywords:** genetically engineered mouse tumor models, KRAS^G12C^ inhibitors, lung adenocarcinoma, RAF1, RAS signaling, tumor resistance

## Abstract

For decades, *KRAS* mutant lung adenocarcinomas (LUAD) have been refractory to therapeutic strategies based on personalized medicine owing to the complexity of designing inhibitors to selectively target KRAS and downstream targets with acceptable toxicities. The recent development of selective KRAS^G12C^ inhibitors represents a landmark after 40 years of intense research efforts since the identification of *KRAS* as a human oncogene. Here, we discuss the mechanisms responsible for the rapid development of resistance to these inhibitors, as well as potential strategies to overcome this limitation. Other therapeutic strategies aimed at inhibiting KRAS oncogenic signaling by targeting either upstream activators or downstream effectors are also reviewed. Finally, we discuss the effect of targeting the mitogen‐activated protein kinase (MAPK) pathway, both based on the failure of MEK and ERK inhibitors in clinical trials, as well as on the recent identification of RAF1 as a potential target due to its MAPK‐independent activity. These new developments, taken together, are likely to open new avenues to effectively treat *KRAS* mutant LUAD.

## Graphical abstract

Four decades after its discovery as a human oncogene, the concept that KRAS was an undruggable target has been challenged. Selective inhibitors against the oncogenic isoform KRAS^G12C^ most frequently observed in lung cancer have been clinically approved, even though tumor resistance to treatment still remains a significant challenge. Nevertheless, clinical results prove that we are witnessing light at the end of the tunnel.

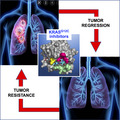

**Abbreviations**
DARPin: designed ankyrin repeat protein
EMT: epithelial‐to‐mesenchymal transition
FDA: U.S. food and drug administration
GAP: GTPase activating protein
GDP: guanosine diphosphate
GEF: guanine nucleotide exchange factor
GEM: genetically engineered mouse
GTP: guanosine triphosphate
KO: knock‐out
LUAD: lung adenocarcinoma
MAPK: mitogen‐activated protein kinase
ORR: overall response rate
PDX: patient‐derived xenograft
PI3K: phosphoinositide 3‐kinase
PROTAC: proteolysis targeting chimera
RTK: receptor tyrosine kinase

## 1 Introduction

Lung cancer is the most lethal tumor type accounting for over 1.7 million deaths worldwide just in 2020 [1]. Despite recent progress in early detection, molecular characterization and development of novel therapeutic strategies, its 5‐year survival rate still remains among the lowest of all cancer types [2]. Nevertheless, the last decade has witnessed major advances in the identification of molecular drivers in most lung cancer types, and in particular in lung adenocarcinomas (LUAD). This information has the potential to increase the accessibility of personalized medicine to a significant number of lung cancer patients. Yet, the key challenge for this type of therapy is to identify targets that, in addition to providing robust therapeutic responses, their inhibition will not cause unacceptable toxicities [3].

One of the most sought‐after therapeutic targets in lung cancer is the *KRAS* oncogene. This oncogene is responsible for at least a quarter of all LUADs. Although *KRAS* was one of the first oncogenes identified in human tumors back in 1982, efforts at developing efficacious inhibitors have failed for almost four decades [4]. However, a recent breakthrough in the development of novel covalent inhibitors has led to the approval of the first selective KRAS inhibitor against one its mutant isoforms: KRAS^G12C^. The FDA granted accelerated approval of sotorasib (AMG510) in May 2021 based on the overall response rate (ORR, 37%) in patients with G12C‐mutated *KRAS* in the CodeBreaK 100 trial [5, 6, 7]. A second KRAS^G12C^ inhibitor, adagrasib (MRTX849), received Breakthrough Therapy Designation from the FDA shortly thereafter based on the results of the KRYSTAL phase I/II trial (ORR 45%) [7, 8, 9].

**Fig. 1:**
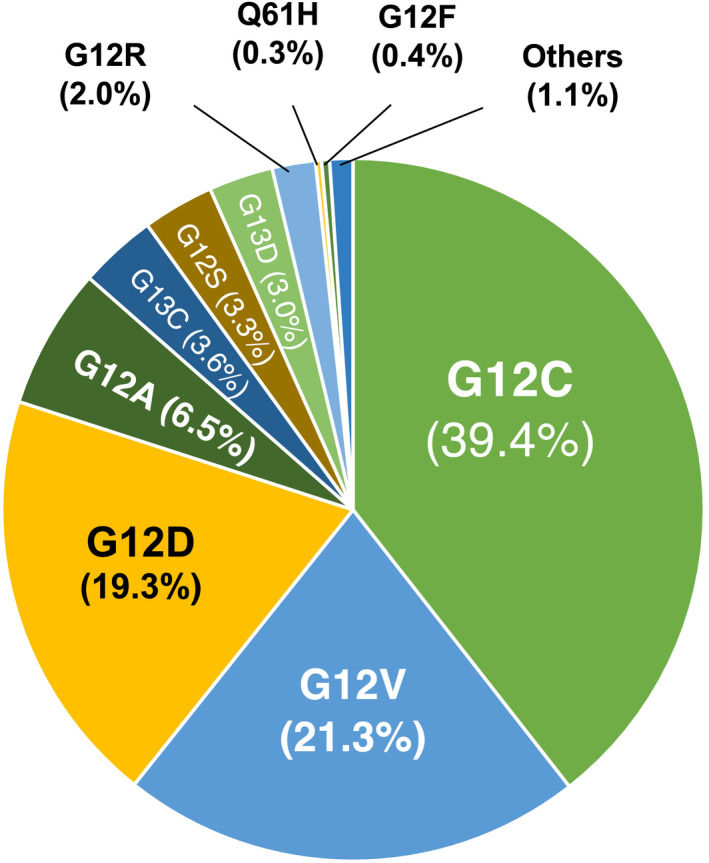
Frequency of *KRAS* mutations in human LUAD. Data were obtained from the Catalogue of Somatic Mutations in Cancer (COSMIC) database of the Sanger Institute v94 (released May 28, 2021).

G12C is the third most frequent *KRAS* mutation, following the G12D and G12V isoforms and represents over 10–12% of all cases. G12C is also the most frequent mutation in LUAD (Fig. 1). In addition, KRAS^G12C^ mutant tumors represent between 3% and 4% of colorectal tumors, and this mutation can also be found in other cancer types albeit at lower frequencies. Thus, the potential benefit from these KRAS^G12C^ inhibitors represents a major breakthrough for the treatment of a significant number of cancer patients. Undoubtedly, the development of these selective KRAS^G12C^ inhibitors marks a historical accomplishment in the long search for therapeutic strategies against *KRAS* mutant tumors and will certainly not be the last.

In this review, we will provide an overview of the biology and functions of KRAS and discuss strategies to treat KRAS‐driven LUADs including those that target KRAS itself as well as alternative strategies involving KRAS signaling focusing on the downstream mitogen‐activated protein kinase (MAPK) pathway and the unique requirement for RAF1. Finally, we will discuss potential strategies to overcome the resistance to the recently developed KRAS^G12C^ inhibitors.

## 2 KRAS biology

KRAS is a small GTPase that comes in two flavors, KRAS4A and KRAS4B, that differ in their carboxy termini due to alternative splicing of the *KRAS* locus. Yet, both protein isoforms along with the highly related RAS family members HRAS and NRAS act as signaling hubs integrating signals from extracellular cues to diverse context‐dependent intracellular programs. It is usually activated by tyrosine kinase receptors (RTKs) such as EGFR, ALK or MET via adapter proteins that activate RAS guanine nucleotide exchange factors (GEFs) including SOS1 and SOS2 that in turn promote replacement of guanosine diphosphate (GDP) with the more abundant guanosine triphosphate (GTP) [10, 11]. GTP‐bound KRAS proteins undergo a conformational change exposing their shared effector binding domain and recruiting a series of effector proteins such as the RAF family of kinases, ARAF, BRAF and RAF1, or the catalytic subunits of the phosphatidylinositol 3‐kinases, p110α, β, δ, and γ, that transmit KRAS signaling to the MAPK and the PI3K‐AKT pathways, respectively [12, 13]. In turn, KRAS signalling is negatively controlled by various GTPase activating proteins (GAPs) that stimulate the limited GTP hydrolysis activity intrinsic to KRAS proteins [14].

The physiological requirements for KRAS have been extensively studied in mouse models. Whereas it is generally accepted that KRAS, like HRAS or NRAS, controls processes such as cell proliferation, migration or differentiation, the *Kras* locus is the only *Ras* locus required for embryonic development [15, 16]. Yet, this requirement cannot be attributed to specific functions of KRAS proteins since replacement of *Kras* by *Hras* sequences supports completely normal development. This requires elimination of the endogenous *Hras* alleles to prevent excess of HRAS proteins which induce cardiovascular pathologies [17, 18]. Finally, systemic elimination of *Kras* alleles from adult mice does not cause obvious detectable defects albeit mice succumb several months earlier than their non‐targeted siblings by, as yet, unidentified toxicities (our unpublished observations).

As indicated above, the *KRAS* locus produces two protein isoforms via alternative splicing, KRAS4A and KRAS4B, that differ only in their hypervariable region at the extreme C‐terminus [10]. Both isoforms are anchored to the plasma membrane through addition of a farnesyl moiety to the cysteine residue that forms part of the CAAX box sequence. Moreover, KRAS4B contains a stretch of basic lysine residues that presumably organizes its localization to distinct nanoclusters. KRAS4A on the other hand uses a hybrid membrane targeting motif consisting of an acylatable cysteine residue as well as a bipartite polybasic region that directs KRAS4A to disorganized domains [19]. Whereas KRAS4A is dispensable for mouse development and homeostasis, the absence of KRAS4B cannot be compensated due to the lower levels of expression of the KRAS4A isoform in most tissues [20, 21]. Despite these generally lower levels of expression, mutations in KRAS4A in the absence of the KRAS4B isoform are still able to induce fully penetrant LUADs in mice [21].

Taken together, these studies indicate that despite substantial differences in membrane attachment and localization, RAS paralogs are surprisingly similar and can, at least to the extend described above, replace each other, a property that, unfortunately, may thwart efforts to specifically target the oncogenic form of KRAS4B. However, these observations suggest that inhibition of KRAS as an anti‐tumor strategy could be well tolerated even if such strategies may target the normal KRAS proteins since they appear to be dispensable for a large time window of adult homeostasis, possibly owed to compensatory roles exerted by HRAS and NRAS.

## 3 *KRAS* mutations in lung cancer

*KRAS* is the most frequently mutated isoform within the RAS family and one of the most frequently mutated oncogenes in human cancer accounting for more than one fifth of all human tumors. Yet, the frequencies of *KRAS* activation vary dramatically between cancer types. For instance, *KRAS* oncogenes are most prevalent in pancreatic adenocarcinoma (90%) and colorectal adenocarcinoma (50%) [22, 23]. In lung tumors, *KRAS* mutations are almost exclusively detected in LUAD (32%) [22]. In this tumor type, close to 90% of all *KRAS* mutations affect codon 12, although codons 13 and 61 are also mutated at lower frequencies (Fig. 1) [23]. The most recurrent mutations in codon 12 cause substitution of the glycine residue with cysteine (40%) or valine (20%) and are usually a consequence of smoking‐induced transversions [24]. Indeed, G12C mutations are found with unprecedented prevalence in current or former smokers [3]. These mutations dramatically impair the GAP‐stimulated GTPase activity of KRAS and change its affinity for downstream effectors to varying degrees [14]. As a consequence, mutant KRAS proteins preferentially bind GTP, although they still cycle between GDP‐ and GTP‐bound states and depend on nucleotide exchange for activation [25]. Moreover, patients with G12C or G12V mutations had worse progression‐free survival than patients with other or no mutation in *KRAS* [26].

## 4 Mouse models of *KRAS* mutant tumors

Introduction of the same *KRAS* mutations present in cancer patients into the genome of genetically engineered mouse (GEM) models is sufficient to induce LUADs that closely mimic those present in human patients once their expression is activated in the lungs of adult mice [27, 28, 29]. Hence, these GEM models have proven particularly useful to validate therapeutic strategies for KRAS‐driven LUAD following pharmacological treatments as well as genetic studies based on gene ablation or inactivation [30]. Although activation of a resident *Kras* oncogene is sufficient to drive lung cancer in these GEM models, most relevant studies have used models that combine *Kras* mutations with mutations or deletion in the p53 tumor suppressor and, to a lesser extent, in LKB1/STK11, another tumor suppressor frequently inactivated in LUAD [31, 32]. Yet, these GEM tumor models develop a few additional mutations, a feature that needs to be taken into account whenever results obtained with these experimental models are extrapolated to those obtained with human patients [33, 34, 35, 36].

Genetically engineered mouse tumor models of *KRAS*/*p53* mutant tumors have been extensively used to validate potential therapeutic targets. Early studies used a strategy in which the target was eliminated at the time of tumor induction [29]. Recently, scientists have developed more sophisticated tumor models in which tumor induction can be temporally and spatially separated from target ablation or inhibition [29]. These models allow the evaluation of the therapeutic effect from target ablation/inhibition in advanced tumors, but also the assessment of potential toxic effects arising from the target under evaluation [37, 38]. Notwithstanding the value of these mouse models, complementary interrogation of the therapeutic potential of targets using other approaches such as patient‐derived xenografts (PDX) is highly desirable.

## 5 KRAS^G12C^ inhibitors

**Fig. 2:**
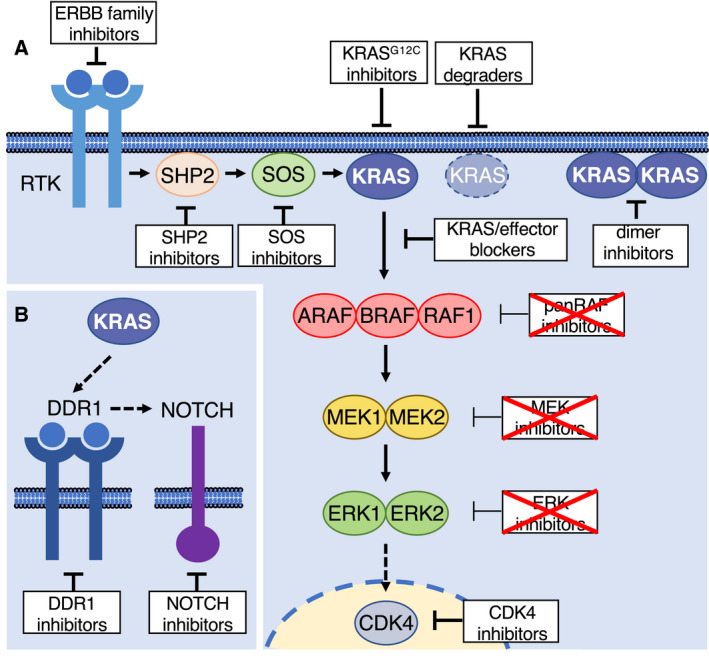
Potential strategies to target KRAS in LUAD. (A) Schematic representation of upstream (tyrosine kinase receptors, RTK, tyrosine phosphatase SHP2, and the guanine nucleotide exchange factor SOS) and downstream (RAF, MEK and ERK kinase families and the cell cycle kinase CDK4) KRAS effectors. Those strategies that interfere with KRAS signaling at different levels are shown as white boxes. White boxes with crossed red lines indicate pharmacological strategies that, so far, have not been approved by the FDA for the treatment of *KRAS* mutant cancers (see reviews [3, 77, 98]). (B) Inhibition of KRAS signaling by DDR1 and NOTCH inhibitors [115].

For decades, approaches to directly target mutant KRAS proteins have failed consistently, ultimately attributing the status of ‘undruggability’ to KRAS. However, the last 5–10 years have witnessed several breakthroughs that dramatically changed this notion and have culminated in the recent approval of sotorasib [5, 6, 7, 39]. In a 2013 landmark study, Shokat and co‐workers identified a previously unrecognized allosteric pocket in the switch II region of the KRAS^G12C^ oncoprotein, a finding that allowed them as well as other investigators to develop covalent inhibitors making use of the reactive cysteine residue in the mutant protein (Fig. 2) [40, 41]. Interestingly, these inhibitors exploit the fact that mutant KRAS^G12C^ proteins are still able to cycle between their active and inactive state and lock them in their GDP‐bound conformation [42, 43].

More recently, several novel inhibitors including sotorasib (AMG510) and adagrasib (MRTX849) have been tested in phase I/II clinical trials [44, 45]. These trials revealed complete or partial responses in 37% of patients with an overall disease control in 80% and have been the basis for the accelerated approval of sotorasib by the FDA [6, 7, 46]. Adagrasib also showed promising results in phase I/II trials (objective response rate 45%; disease control rate 96%) resulting in Breakthrough Therapy Designation by the FDA [8]. Moreover, given the highly selective mechanism of action, KRAS^G12C^ inhibitors present a favorable safety profile and clinical trials only revealed minor adverse events [5, 46].

## 6 Resistance to KRAS^G12C^ inhibitors

Unfortunately, the majority of patients treated with these inhibitors experienced tumor progression after a few months of treatment [47, 48]. Recent studies have revealed that some of these resistant tumors contain additional mutations in the *KRAS* ^G12C^ oncogene itself such as Y96D, a mutation that affects binding of adagrasib to its pocket in switch II [49]. The same study also postulated that a different class of KRAS^G12C^ inhibitors targeting GTP‐bound active KRAS^G12C^ in a complex with cyclophilin A could overcome resistance due to mutations in the adagrasib binding pocket. An *in vitro* screen for mutations that confer resistance to adagrasib or sotorasib also revealed drug concentration–dependent secondary *KRAS* mutations including Y96D [50].

A more recent study also identified a series of potential resistance mechanisms in a cohort of patients treated with adagrasib that included amplification of the *KRAS* ^G12C^ allele, additional activating mutations in codons 12, 13 or 61 of *KRAS* or a variety of bypass mechanisms involving RTK or MAPK effector activation [9]. Some patients in this study also developed resistance via mutations in the switch II pocket such as Y96C or H95D/Q/R. Yet, these additional mutations are present in a limited number of cells, thus suggesting that they may not be the direct or at least the immediate cause of the resistance to G12C inhibitors [9, 49].

Other potential mechanisms of resistance that have been detected in cell lines include tyrosine kinase receptor reactivation and KRAS bypass [51], re‐expression of inhibitor insensitive GTP‐bound KRAS^G12C^ and AURKA overexpression [52] or feedback re‐activation of MAPK signaling [44]. For identification of potential resistance mechanisms in a systematic fashion, GEM models expressing KRAS^G12C^ will be particularly useful ([53] and our unpublished data).

Finally, no additional mutations have been identified in a significant percentage of resistant tumors. Yet, non‐genetic resistance mechanisms have also been described and may, at least on some occasions, account for resistance to KRAS^G12C^ inhibitors. Indeed, several studies proposed the intrinsic plasticity of heterogeneous tumor cells as a potential underlying cause of resistance. For instance, epithelial‐to‐mesenchymal transition (EMT) has been shown to cause resistance to sotorasib in lung cancer cell lines [54]. Interestingly, KRAS independence had already been linked to EMT in lung cancer cell lines before [55]. A more recent study identified increased FGFR and AXL signaling as mechanisms of resistance specifically in mesenchymal cell types when treated with KRAS^G12C^ inhibitors [56]. Moreover, Awad et al. [9] identified a histologic transformation of LUAD to squamous cell carcinomas in two patients treated with adagrasib. These observations indicate that the plasticity of epithelial tumor cells may, at least to some extent, contribute to non‐genetic mechanisms of resistance.

## 7 Overcoming resistance to KRAS^G12C^ inhibition

It is generally accepted that combination therapies could maximize the therapeutic efficacy of KRAS^G12C^ inhibitors ultimately resulting in prevention, or at least delay, of the appearance of resistance. Indeed, combining sotorasib or adagrasib with anti–PD‐1 immunotherapy was more effective than either treatment alone in syngeneic mouse models [44, 57]. In particular, KRAS^G12C^ inhibition with sotorasib caused a pro‐inflammatory microenvironment accompanied by expression of cytokines such as *Cxcl10* or *Cxcl11* and increased infiltration of CD8^+^ T cells, suggesting that T cell activation following treatment with α‐PD1 antibodies is a prerequisite for the synergistic activity [44]. Comparable results were obtained when adagrasib was combined with α‐PD1 antibodies, leading to durable and complete tumor regression rates [57]. These observations are in agreement with a series of studies highlighting the immunosuppressive properties of mutant KRAS through a variety of mechanisms including stimulation of PD‐L1 expression [58], promotion of an inflammatory microenvironment [59] or interference with antigen presentation [60]. Taken together, accumulating evidence suggests that KRAS^G12C^ inhibition may have profound consequences on the tumor microenvironment making these inhibitors prone to be combined with immunotherapeutic approaches.

KRAS^G12C^ inhibitors have also been combined with a battery of compounds in *in vitro* assays. For instance, combining KRAS^G12C^ inhibitors with IGF1R and mTOR inhibitors led to efficient responses in lung cancer cell lines [61]. Similar results were obtained in *KRAS* mutant cells that feedback‐activated mTORC2 after treatment with sotorasib [62]. Moreover, adagrasib was found to potently synergize with the ERBB family inhibitor afatinib (see below) or the CDK4/6 inhibitor palbociclib (see below) (Fig. 2) [45]. Notably, the synergistic effect of KRAS^G12C^ inhibition with the ERBB family inhibitor afatinib was linked to a more epithelial cell subtype whereas those resistant cells that displayed a more mesenchymal phenotype could be targeted with AXL inhibitors [56]. Inhibitors of the tyrosine kinases MET, SRC or FGFR were also found to increase the efficacy of KRAS^G12C^ inhibition [43, 56]. Yet, it was suggested that the synergistic effect of RTK inhibition may be limited by the activation of selective RTKs in each tumor, thus supporting a more personalized approach to maximize the effect of KRAS^G12C^ inhibition [25].

Since current KRAS^G12C^ inhibitors such as sotorasib or adagrasib covalently bind to GDP‐loaded KRAS, enhancing the probability of GDP binding could also improve the therapeutic impact of these compounds. For instance, the phosphatase SHP2 has been shown to play a vital role in RTK‐mediated KRAS activation, and its inhibition efficiently reduced KRAS GTP‐loading [63, 64]. SHP2 is thought to act upstream of the RAS GEFs SOS1/SOS2, and its inhibition may facilitate inactivation of mutant KRAS [65]. As a consequence, recent evidence illustrated that SHP2 inhibitors efficiently enhanced the therapeutic effect of KRAS^G12C^ inhibition, but also prevented feedback activation of the MAPK pathway upon KRAS inhibition in cell lines (Fig. 2) [51, 52, 56, 66]. Currently, several SHP2 inhibitors are being evaluated in clinical trials alone or in combination with KRAS^G12C^ inhibitors [67]. Likewise, direct inhibition of the exchange factor SOS1 has been proposed to impact on KRAS GTP‐loading potentially increasing the efficacy of KRAS^G12C^ inhibitors [68]. Recently, several inhibitors that disrupt the SOS1‐KRAS interaction have been developed that synergized with KRAS^G12C^ inhibitors in cell lines [69, 70].

## 8 Degrading KRAS, an alternative approach?

KRAS^G12C^ inhibitors will undoubtedly have a profound impact on the treatment of KRAS‐driven lung cancer. However, as indicated in the previous chapters, second‐site mutations in *KRAS* may dramatically limit the efficacy of KRAS^G12C^ inhibitors in the clinic. To avoid this mechanism of resistance, targeted protein degradation strategies such as those mediated by the recently developed proteolysis targeting chimeras (PROTACs) could be a valuable approach (Fig. 2) [71]. PROTACs based on covalent KRAS^G12C^ inhibitors have been developed, but they unfortunately showed poor antiproliferative effects due to their limited ability to induce repeated cycles of protein degradation [72, 73]. Thus, PROTACs based on future non‐covalent inhibitors represent a more promising strategy to target KRAS oncoproteins.

However, PROTACs are not the only way to selectively degrade KRAS. For instance, a recent study identified a monobody that selectively bound to KRAS^G12V^ and KRAS^G12C^ and prevented effector binding [74]. Fusion of this monobody to the E3 ubiquitin ligase VHL caused selective degradation of KRAS oncoproteins, indicating that efficient degradation of KRAS mutants is achievable. Similarly, a KRAS‐specific DARPin, a type of therapeutic agent derived from natural ankyrin repeat proteins that has the potential to overcome some of the limitations of monoclonal antibodies, has been developed that also caused selective degradation of KRAS when fused to the VHL E3 ligase and inhibited cell proliferation in cells carrying a mutant *KRAS* gene [75]. Although still far from being applicable to patients, these studies emphasize the potential benefit of inducing KRAS degradation.

## 9 Blocking KRAS–effector interactions

Blocking the interaction of KRAS oncoproteins with their effectors may also be a promising strategy for the treatment of *KRAS* mutant lung tumors (Fig. 2) [76]. As indicated above, there is ample evidence that preventing the activation of KRAS signaling pathways can interfere with KRAS‐driven tumorigenesis [12, 77]. For instance, rigosertib has been discovered as a RAS‐mimetic that is thought to mimic the RAS‐binding domain of several RAS effectors leading to the effective sequestration of effectors such as the RAF kinases, RalGDS or the catalytic p110 isoforms of PI3K [78]. Although rigosertib is currently undergoing clinical evaluation in a phase 3 trial, substantial controversy exists as to its anti‐tumor mechanism. A recent study has proposed that rigosertib might exert its anti‐tumor activity as a microtubule‐destabilizing agent, an observation that has been questioned by the original authors [79, 80]. Notwithstanding, the discovery of rigosertib exemplifies that small molecule compounds can block the interaction between KRAS and its effectors. Another rationally designed compound, termed Abd‐7, was designed via sequential epitope determination with antibody fragments and was shown to block the binding of mutant KRAS proteins to its effectors [81].

The usefulness of targeting KRAS effector interactions had also been demonstrated by *in silico* screening. For instance, the compounds Kobe0065 and Kobe2602 blocked the effector interaction of GTP‐loaded HRAS and KRAS proteins [82]. Although these compounds also bind to a variety of other small GTPases, which may lead to elevated toxicity, they may nevertheless serve as lead scaffolds for the development of selective compounds to block KRAS signaling. The non‐steroid anti‐inflammatory drug sulindac and several of its analogues were also reported to interfere with the RAS/RAF interaction and induce anti‐tumor effects, although some of these observations remain controversial [76, 83]. In addition, the feasibility of interfering with effector binding was demonstrated by engineering a cell‐permeable RAS‐binding domain polypeptide that achieved potent and selective anti‐tumor effects in *KRAS* mutant cell lines [84].

Pioneering work by Downward and colleagues has also shown tumor regression upon inhibition of the interaction between KRAS oncoproteins and the p110α subunit of PI3K [85]. However, these studies were carried out over short periods of time, and it is possible that significant toxicities may also appear upon longer treatments. Thus, it is possible that the failure of PI3K inhibitors observed in multiple clinical trials against *KRAS*‐mutant tumors may be attributed to the toxic consequences derived from blocking this pathway. Finally, it has been proposed that KRAS may form dimers or some other high‐order structures [86]. Hence, interfering with dimerization could represent another strategy to block its oncogenic activity [87]. In conclusion, blocking the interactions of KRAS oncoproteins with their main downstream effectors represents a promising strategy that may be particularly useful to overcome resistance to KRAS^G12C^ inhibitors.

## 10 Inhibitors of the MAPK pathway

For more than a decade, the pharmaceutical industry has focused research efforts on the development of inhibitors against the RAF, MEK and ERK kinases [88, 89]. Considerable efforts were also allocated to develop inhibitors of the PI3K p110α and β subunits as well as against the AKT1 kinase [90]. Indeed, this strategy was fueled by the success in the development of selective inhibitors against the *BRAF* ^V600E^ oncogene present in melanoma. The rapid development of a very effective inhibitor, vemurafenib [91], led to the idea that similar strategies might be equally successful in blocking KRAS oncogenic signaling.

Unfortunately, and despite extensive efforts, none of the RAF, MEK or ERK kinase inhibitors developed thus far have been approved for the treatment of *KRAS* mutant tumors. Only the MEK inhibitor selumetinib has been approved for the treatment of pediatric tumors induced by *NF1* mutations [92]. Likewise, another MEK inhibitor widely used in GEM models, trametinib, has been approved for the treatment of metastatic melanoma in combination with the BRAF^V600E^ inhibitor dabrafenib [93]. Yet, the use of this drug combination is rather limited as a result of its high toxicity. So far, no ERK inhibitor has gone beyond phase II in clinical trials. Likewise, panRAF or other RAF inhibitors of various classes (e.g. paradox breakers, etc.), have failed to show significant anti‐tumor activity at acceptably tolerated doses [89].

The reason for the unacceptable toxicities of MEK and ERK inhibitors is still a matter of debate. Most studies are focusing on the prevalence of feedback circuitries that prevent effective MEK or ERK kinase inhibition [94]. Yet, it is also possible that these signaling pathways are essential for normal homeostasis [89]. Thus, any significant tampering with their signaling activity may lead to unacceptable toxicities in a manner not too different from that observed with classical chemotherapy compounds that target essential cellular activities. Indeed, genetic interrogation of the role of each of the nodes of the MAPK pathway in GEM models has revealed that ablation of either MEK1/2 or ERK1/2 kinases resulted in the rapid death of the mice due to severe toxicity in their intestinal crypts [95]. Similar results have been observed upon concomitant ablation of the three members of the RAF family of kinases (our unpublished observations). On the other hand, genetic elimination of individual members of each of these kinase families did not display significant toxicities [95]. Unfortunately, such approaches did not induce detectable anti‐tumor activity, with the exception of RAF1 (see below). These results suggest that the failure of drugs targeting the MAPK pathway in clinical trials for *KRAS* mutant lung cancer could, at least to some extent, be explained by the essential role of the MAPK pathway in normal homeostasis [89].

In spite of these setbacks, several new compounds targeting these kinases are currently under clinical evaluation [77]. It is possible that strategies combining inhibitors of the MAPK pathway at different levels could be more effective, as the combined use of lower concentrations of each individual drug may achieve better anti‐tumor effects with more acceptable toxicities than the use of single MAPK pathway inhibitors [96]. Likewise, MAPK pathway inhibitors may be combined with other unrelated inhibitors, and this concept has yielded a multitude of potential drug combinations, some of which have shown promising results in preclinical studies of KRAS‐driven tumors. Since these studies have already been the subject of several recent reviews, they will not be discussed here [77, 89, 97, 98, 99, 100]. In summary, ample experimental evidence indicates that activation of the MAPK pathway is essential for KRAS‐driven LUAD, but at the same time it might be equally relevant for normal homeostasis, thus posing a substantial barrier to the treatment of cancer patients.

## 11 RAF1, a key player in *KRAS* mutant tumors

Despite the above limitations in the use of MAPK pathway inhibitors, studies using GEM tumors models have identified an unexpected requirement for RAF1 in KRAS‐driven LUAD. Early studies revealed that tumor initiation was completely prevented by ablation of the *Raf1* gene, but not by ablation of the other RAF family members *Braf* [95, 101] or *Araf* (our unpublished observations). Notably, the requirement for RAF1 was not limited to tumor initiation, a usually less stringent condition, but extended to tumor progression and maintenance, thus indicating that RAF1 plays a crucial role in KRAS‐driven LUAD [38]. Indeed, RAF1 ablation in *Kras*/*Trp53* mutant tumor‐bearing LUADs led to significant regressions in more than two thirds of the tumors, including some complete regressions [38].

**Fig. 3:**
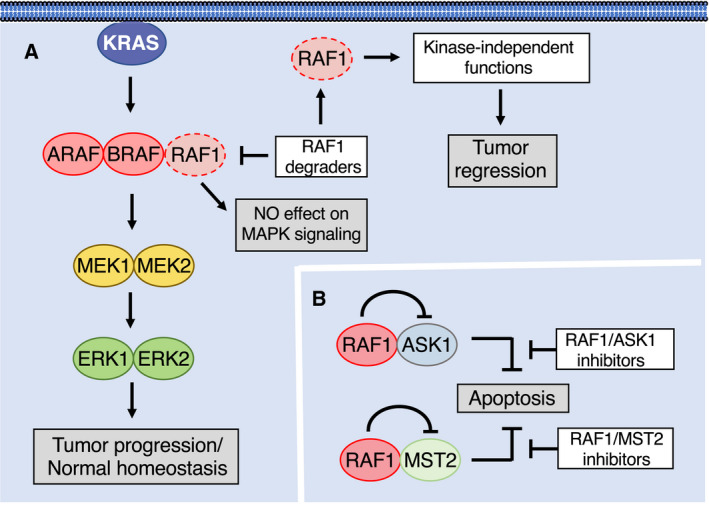
Potential strategies to target RAF1 in KRAS‐driven LUAD. White boxes indicate conceivable therapeutic options and grey boxes the consequences on tumor growth. (A) RAF1 ablation or its degradation has no effect of MAPK signaling or normal homeostasis. However, it causes regression of LUAD via kinase‐independent functions. (B) RAF1 blocks apoptosis by activating ASK1 and MST2. Hypothetical RAF1/ASK1 or RAF1/MST2 inhibitors are predicted to liberate ASK1 and MST2 from the inhibitory effect of RAF1 kinase–independent activity to induce apoptosis.

Surprisingly, acute elimination of RAF1 expression in lung tumors did not affect the activity of the MAPK pathway, suggesting that the requirement for RAF1 in *KRAS* mutant tumors is unrelated to its role within the MAPK pathway (see below, Fig. 3A). More importantly, in contrast to the combined elimination of all three RAF family members, systemic elimination of RAF1 expression did not cause detectable toxicities in adult mice, indicating that RAF1 could be an effective target for tumors that either have developed resistance to sotorasib or adagrasib, or for those that do not carry a targetable G12C mutation.

Selective targeting of RAF1 will be a considerable challenge. Despite being a kinase, the role of RAF1 in driving *Kras* mutant tumors is not mediated through its catalytic activity (Fig. 3) [102]. Mice expressing a kinase‐inactive RAF1 isoform (RAF1^D468A^) developed LUAD with the same incidence and latency as those mice expressing the wild‐type protein. A second strain expressing an independent RAF1 kinase dead isoform (RAF1^K375M^) displayed a somewhat limited tumor burden. Yet, these observations were partially attributed to the instability of the RAF1^K375M^ kinase dead isoform. This unanticipated result suggests that pharmacological efforts to block RAF1 activity will have to be based on strategies other than kinase inhibitors.

### 11.1 Kinase‐independent functions of RAF1

Although RAF1 is a protein kinase known to phosphorylate substrates such as MEK1, MEK2, or even additional proteins including RB, MEKK1, IκB and BAD, further kinase‐independent roles have been described [89, 103]. For instance, RAF1 was shown to interact with ROKα (ROCK2) and inhibit the ROKα kinase activity, and this effect was independent of the catalytic activity of RAF1 [104]. This interaction played a role in K5‐SOS‐F‐driven squamous skin tumor growth, as ablation of RAF1 expression from established tumors caused their regression in a ROKα‐dependent manner [105].

Several studies have demonstrated that RAF1 has anti‐apoptotic activity, and this activity was thought to be mediated by the inactivation of the pro‐apoptotic kinases ASK1 and MST2 in a catalysis‐independent manner (Fig. 3B) [106, 107]. Consistent with these findings, mice lacking *Raf1* succumbed during embryonic development as a result of apoptosis induction in the liver as well as several tissues, which suggests that suppression of apoptosis plays a relevant role under physiological conditions [108]. In agreement with these observations, ablation of RAF1 expression from KRAS‐driven lung tumors also caused a strong apoptotic response [38]. Indeed, depletion of either ASK1 or MST2 was sufficient to revert the anti‐proliferative effect of RAF1 depletion in human tumor cell lines and PDX models [102]. Moreover, heart dysfunction and cardiac fibrosis present in muscle‐specific RAF1 knock‐out (KO) mice were rescued by genetic deletion of ASK1 [109]. Interestingly, apoptosis induction upon RAF1 elimination also involves the FAS receptor, and tempering with FAS activation prevented apoptosis in the liver of RAF1 KO embryos allowing them to develop to term [110]. Why RAF1 has evolved to play these additional roles outside of the MAPK pathway is currently unknown, but may be a consequence of a functional separation from the more ancestral BRAF isoform [111].

These observations open the door to the development of therapeutic strategies against *KRAS*‐mutant tumors that either involve stimulation of the pro‐apoptotic activities of ASK1 and/or MST2 or prevent ASK1 and/or MST2 inhibition by RAF1 (Fig. 3B). Yet, such strategies will require a more profound understanding of the mechanism of action of these pro‐apoptotic kinases. Likewise, it will be essential to define the structural details as of how the interaction of RAF1 results in the functional inactivation of these pro‐apoptotic kinases.

Selective induction of the effective RAF1 degradation thus emerges as the most promising strategy to pharmacologically block RAF1 activity in *KRAS* mutant LUAD [112]. The use of PROTACs to degrade RAF1 will require the identification of unique pockets within RAF1. The use of RAF1 kinase inhibitors known to bind to its catalytic site is certainly an option. Yet, not all RAF1 binders might be able to bring E3 ligases in proximity to those ubiquitinable lysine residues essential to trigger its degradation. A better understanding of the full structure of RAF1 might unveil additional vulnerabilities that might be utilized to induce its efficient degradation either via PROTACs or other degradation strategies.

### 11.2 RAF1 inhibition: combination strategies

Unlike human LUADs, lung tumors developing in *Kras*/*Trp53*‐driven GEM tumor models exhibit a much more limited tumor burden. Hence, the significant levels of tumor regression observed upon RAF1 ablation are likely to be more limited upon RAF1 degradation in human patients. Therefore, it is important to identify additional targets that may cooperate with RAF1 degradation in a clinical scenario. One of such potential targets is the cell cycle kinase CDK4. Previous studies from our laboratory have shown that expression of a *Kras* oncogene in lung cells lacking CDK4 expression–induced senescence [113]. No such effect was observed in mice lacking the related CDK2 or CDK6 cell cycle kinases. To interrogate the potential role of blocking CDK4 kinase activity in LUAD, we generated a mouse strain that expressed a kinase‐inactive CDK4^K35M^ protein, thereby mimicking the activity of an optimal CDK4 inhibitor [114]. Combined expression of this kinase dead isoform with RAF1 ablation significantly improved its therapeutic effect in mice carrying *Kras*/*Trp53*‐driven LUADs without increased toxicity. Indeed, we observed complete regressions in a quarter of the tumors. Moreover, none of the remaining tumors displayed tumor progression. Thus, a combination therapy consisting of RAF1 degradation and CDK4 kinase inhibition could be effective in patients with *KRAS* mutant lung tumors [114]. Interestingly, CDK4/6 inhibitors also synergized with KRAS^G12C^ inhibitors in *in vitro* studies [45].

Other targets may also be effectively combined with RAF1 elimination. For instance, combined inhibition of DDR1 and NOTCH signaling was proposed to be an effective therapeutic strategy for *Kras*/*Trp53*‐driven lung tumors with no signs of excessive toxicity (Fig. 2) [115]. Therefore, combining RAF1 inhibition with either DDR1 and/or NOTCH blockade may result in synergistic effects that will end up providing alternative solutions for patients carrying *KRAS* mutant tumors. Finally, mutant KRAS was shown to activate the ERBB network through a feed‐forward loop and inhibition of ERBB family receptors with drugs such as afatinib affected the growth of KRAS‐driven tumors [116, 117]. These results suggest that ERBB family inhibitors could also synergize with RAF1 degradation, as already demonstrated with KRAS^G12C^ inhibitors [44]. We also anticipate that RAF1 degradation could synergize with PD‐1 blockage given the increase in numbers of tumor‐infiltrating CD8^+^ T cells upon genetic RAF1 ablation alone [38]. RAF1 degradation may also synergize with SHP2 and SOS1 inhibitors due to their potential to lower the activation state of KRAS.

Finally, an interesting issue raised by our observations is whether RAF1 degradation would be equally effective in tumors with *KRAS* mutations other than the KRAS^G12V^ mutation. For instance, KRAS^G12V^ oncoproteins appear to have a higher affinity for RAF1 than other mutant variants [14]. In contrast, KRAS^G12C^ binds RAF1 with affinities close to wild‐type KRAS. Whether this disparity in RAF1 binding affinity may translate into different outcomes upon RAF1 degradation remains to be determined.

## 12 Identification of novel vulnerabilities via proteogenomic studies

In addition to the above‐described strategies to target *KRAS*‐mutant lung cancer, it is particularly relevant to identify additional targets that could contribute to the treatment of patients with lung cancer. To this end, a series of recent studies has employed sophisticated proteomic or proteogenomic approaches to identify novel vulnerabilities. For instance, multi‐omic analyses of a large number of lung tumor specimens revealed some highly *KRAS* mutant‐selective phosphorylation events such as those in SOS1 or DNMBP, again strengthening the idea that inhibition of SOS1 could be a promising strategy for these tumors [118]. Another study that combined proteomics with genetic interactions mapping identified novel KRAS interactors of which at least two, RAP1GDS1 and RHOA were selectively required for KRAS‐mutant LUAD [119]. Other approaches have revealed new drug combinations that were specifically vulnerable to *KRAS*‐mutant tumors such as inhibition of DOTL1 and SHP2 [120] or WEE1 and ERK [121]. Taken together, these studies highlight the fact that proteomic or proteogenomic analyses can reveal novel vulnerabilities which may prove particularly useful in those tumors that do not respond to any of the strategies described above or in cases of resistance to targeted therapies.

## 13 Future perspectives

Personalized medicine requires the development of highly selective cancer therapies that cause little or no toxicity. Until very recently, *KRAS* mutant cancer never benefitted from these strategies due to the lack of suitable inhibitors. However, the recent development of KRAS^G12C^ inhibitors has represented a major breakthrough in the path towards the development of additional personalized therapies for KRAS‐driven cancers. The approval of the first KRAS^G12C^ inhibitor is expected to have profound consequences on the management of patients. However, experience from both clinical trials and *in vitro* experiments indicates that resistance to KRAS^G12C^ inhibitors occurs rapidly when they are used as single agents. Thus, it is urgently required to understand the basic mechanisms of resistance to be able to develop new drugs or drug combinations that overcome the observed tumor resistance.

G12C mutations account for almost 40% of all *KRAS* mutant LUADs as well as 3–4% of colorectal tumors. Yet, it is essential to develop selective inhibitors against the other mutations, mainly G12D and G12V, or, even better, pan‐KRAS inhibitors capable of blocking all mutant oncoproteins. Currently, there are also efforts to develop selective inhibitors against the G12R mutation frequent in PDAC, the tumor type with the highest frequency of *KRAS* mutations and one of the tumors with worse prognosis. Strategies to block KRAS signaling should also be actively pursued providing that they do not block essential signaling mechanisms. The development of degron and other strategies, recently reviewed in references [71], [112] and [122] should facilitate the generation of more selective and robust inhibitors. Among these approaches, eliminating the expression of RAF1 is a promising therapeutic option. Although there is no drug that fulfills this task to date, loss of RAF1 expression is well tolerated in adult mice [38], thus indicating that elimination of RAF1 may synergize with inhibition of KRAS, causing only limited toxicities. Clearly, the recent development of selective KRAS^G12C^ inhibitors has completely changed the notion of ‘undruggability’ for KRAS proteins, thus opening new avenues that will allow personalized medicine to effectively treat *KRAS* mutant LUAD.

## Conflict of interest

The authors declare no conflict of interest.

## Author contributions

MD and MB contributed equally at writing the manuscript.
